# Network analyses reveal new insights into the effect of multicomponent Tr14 compared to single-component diclofenac in an acute inflammation model

**DOI:** 10.1186/s12950-023-00335-0

**Published:** 2023-03-27

**Authors:** Matti Hoch, Suchi Smita, Konstantin Cesnulevicius, Myron Schultz, David Lescheid, Olaf Wolkenhauer, Shailendra Gupta

**Affiliations:** 1 Department of Systems Biology and Bioinformatics, University of Rostock, Rostock, 18055 Germany; 2 Heel GmbH, Baden-Baden, 76532 Germany; 3 Leibniz-Institute for Food Systems Biology at the Technical University of Munich, Freising, 85354 Germany; 4 Stellenbosch Institute of Advanced Study, Wallenberg Research Centre, Stellenbosch University, Stellenbosch, 7602 South Africa

**Keywords:** Acute inflammation, Inflammation resolution, Multitarget drugs, Network modeling, Network pharmacology, Systems biology, Traumeel, Diclofenac

## Abstract

**Background:**

Modifying the acute inflammatory response has wide clinical benefits. Current options include non-steroidal anti-inflammatory drugs (NSAIDs) and therapies that may resolve inflammation. Acute inflammation involves multiple cell types and various processes. We, therefore, investigated whether an immunomodulatory drug that acts simultaneously at multiple sites shows greater potential to resolve acute inflammation more effectively and with fewer side effects than a common anti-inflammatory drug developed as a small molecule for a single target. In this work, we used time-series gene expression profiles from a wound healing mouse model to compare the effects of Traumeel (Tr14), a multicomponent natural product, to diclofenac, a single component NSAID on inflammation resolution.

**Results:**

We advance previous studies by mapping the data onto the “Atlas of Inflammation Resolution”, followed by *in silico* simulations and network analysis. We found that Tr14 acts primarily on the late phase of acute inflammation (during resolution) compared to diclofenac, which suppresses acute inflammation immediately after injury.

**Conclusions:**

Our results provide new insights how network pharmacology of multicomponent drugs may support inflammation resolution in inflammatory conditions.

**Supplementary Information:**

The online version contains supplementary material available at 10.1186/s12950-023-00335-0.

## Background

Acute and chronic inflammation are dynamic, multifactorial processes. They are controlled by non-linear feedback and feedforward regulatory loops offering multiple potential spatiotemporal targets for therapeutic interventions [1–3]. To better understand the non-linear relationship among immune cell types, signaling, and regulatory molecules associated with the onset, transition, and resolution of acute inflammation, we developed a comprehensive “Atlas of Inflammation Resolution (AIR)” (https://air.bio.informatik.uni-rostock.de). We developed the AIR as a research tool to identify and prioritize therapeutic targets, analyze the impact of molecular perturbations on acute inflammatory processes and phenotypes, and understand the mode of action of drug candidates [4].

Current scientific evidence indicates that the resolution of acute inflammation is an active process, proposing that its stimulation could be a novel therapeutic approach. While drug discovery remains highly focused on the one-molecule, one-target approach, clinical evidence often demonstrates limited effectiveness of single-component therapy [5, 6]. By comparison, drugs targeting multiple players involved in different biochemical pathways can overcome adaptive resistance [6–10]. Similarly, medicines containing several active ingredients, a feature of many natural products, could potentially be more effective for treating multifactorial diseases [11–13]. How natural product-based drugs work is generally determined using extensive screening processes. These include time-consuming cell-based reporter or cell viability assays. Unfortunately, such techniques may not fully reveal a drug’s molecular mechanisms of action [14, 15]. Identifying suitable therapeutic drug combinations increasingly involves adopting a systems biology approach to elucidate their targets and mode of action [10, 16–20]. This approach allows researchers to study spatial and temporal cellular functions, feedback mechanisms, and dynamic molecular interaction. At a large scale, molecular interaction networks integrate hundreds to thousands of interactions linked to specific disease processes or phenotypes commonly referred to as disease maps [21–23].

In this study, we employed a previously published time-series transcriptomics dataset from an in vivo murine model of the cutaneous wound healing [24–27]. The source material included responses to treatment with the single-component drug diclofenac and multicomponent natural product Traumeel (Tr14) [27]. Diclofenac is a non-steroidal anti-inflammatory drug (NSAID) known to inhibit the synthesis of prostanoids such as prostaglandin-E2 (PGE2), prostacyclins, and thromboxanes by blocking both cyclooxygenase 1 (COX-1) and cyclooxygenase 2 (COX-2) enzymes [28, 29]. In contrast, Tr14 has been shown to regulate several pathways associated with the resolution of acute inflammation, including apoptosis, leukocyte migration, and angiogenesis in the in vivo murine wound healing models [24, 27]. Tr14 positively impacts the synthesis of specialized pro-resolving lipid mediators (SPMs) in human monocyte-derived macrophages by enhancing efferocytosis and SPM production in a zymosan-induced mouse model [30]. Additionally, in previous double-blind randomized controlled trials (RCT), Tr14 has been shown to reduce pain, one of the hallmarks of acute inflammation, after musculoskeletal injury [25, 26, 31].

The current study adds to this previous work by using the AIR molecular interaction network to investigate the impact of differential expression on inflammatory pathways and cell types. The AIR facilitates and enhances the transcriptomics analysis by (i) filtering genes directly connected to inflammatory processes; (ii) intuitively visualizing spatiotemporal differences between the treatment effects; (iii) inferring the direction and strength of enriched processes; and (iv) generating subnetworks of causal interactions that link differentially expressed genes with inflammatory phenotypes. These advancements enable more detailed insights into the mode of action of both treatments than from the data alone. Using a systems biology approach with the AIR, we compared how these two fundamentally different treatments (multicomponent and single-component) differentially modulate molecular and cellular profiles to better understand their impact on acute inflammation and its resolution.

## Results

### Tr14 and diclofenac have different temporal expression profiles of genes unique to the acute inflammation signaling landscape

In the publicly available time-series transcriptomics data from the wound healing mouse model, six outliers, three in the control (saline injection) group (12 h, 72 h, and 96 h), two in the ‘Tr14’ group (12 h and 120 h), and one in the ‘diclofenac’ group (96 h) were identified. After removing these outliers, we observed an increased number of DEGs in the differential analysis of treatment groups compared to their respective control groups (Figures S1 – S5).

**Fig. 1:**
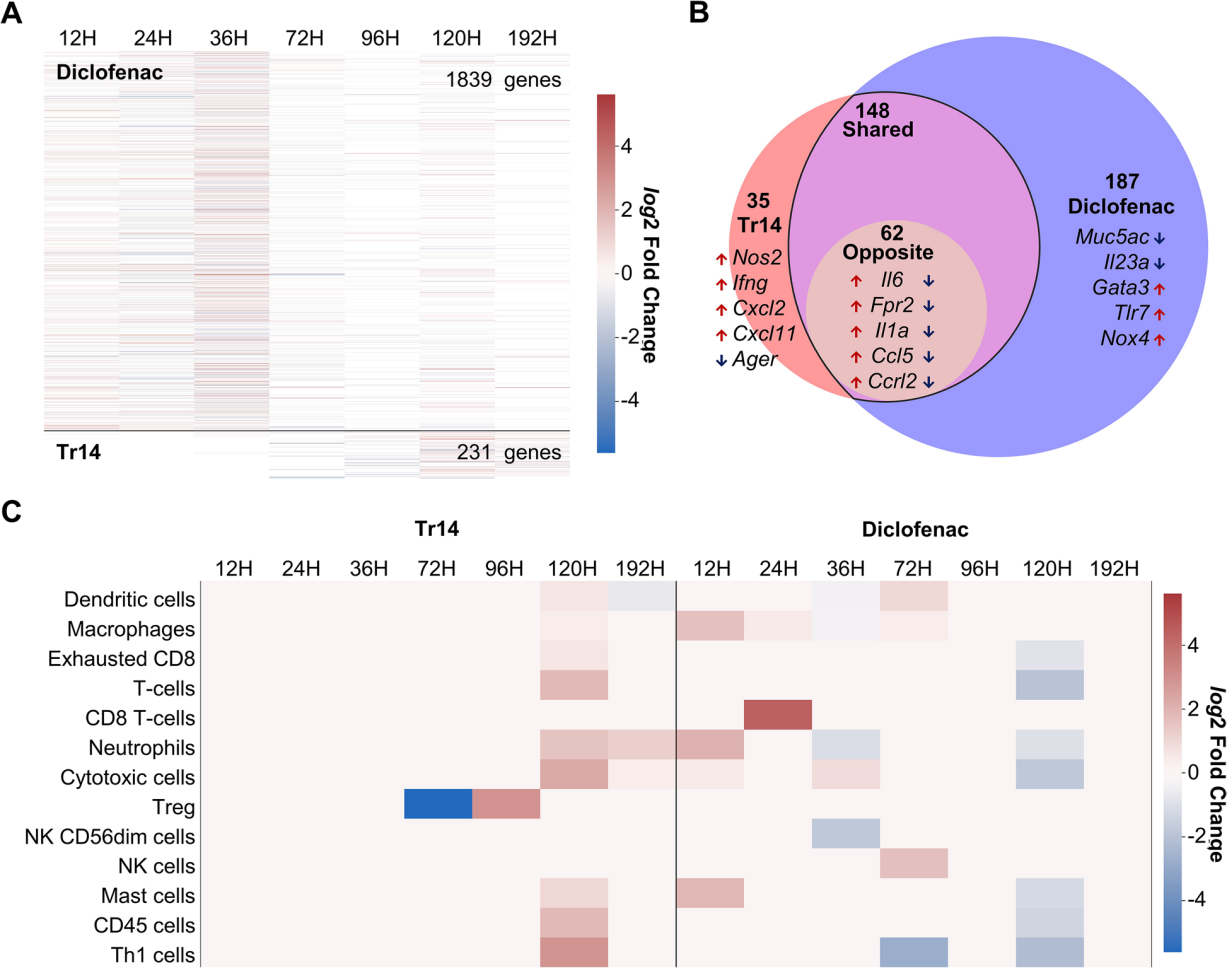
**A** Heat map highlighting expression profile (Log2 FC) of the unique DEGs in Tr14 and diclofenac treatments compared to their respective control at various time points in the AIR Molecular Interaction Map. **B** Venn diagram showing unique DEGs present in the AIR submaps, which are directly involved in regulating acute inflammatory processes/phenotypes. Only the top 5 genes based on the highest absolute log2FC value are labeled in each group (Tr14, diclofenac, and shared DEGs with opposing expression profiles in both treatment groups). **C** Immune cell profiling using the aggregated log twofold change expression profile of marker genes in Tr14 vs. saline control and diclofenac vs. placebo control. The red color indicates an increase, whereas the blue indicates a decrease in the cell population at the given time point shown in the column header

In the AIR MIM, 1839 unique DEGs associated with the regulation of acute inflammatory processes and phenotypes were found in diclofenac and 231 in Tr14 treatment groups compared to placebo controls at all analyzed time points (Fig. 1A) (Supplementary Excel file 2). Most of the DEGs were identified at the early time point (before 36 h) in the diclofenac treatment group, while for Tr14, most appeared after 72 h. DEGs present only in the AIR submaps directly linked to acute inflammation initiation, transition, resolution, and homeostasis were further filtered (Fig. 1B). This revealed 187 unique DEGs in the diclofenac treatment group that were not differentially expressed at any time point in the Tr14 group compared to placebo controls. Similarly, 35 unique DEGs in the Tr14 treatment group were encountered at all analyzed time points. By comparison, 148 common DEGs were identified following both treatments. Among them, 62 genes were differentially expressed in opposite directions (solely upregulated in Tr14 but downregulated in diclofenac compared to their respective control or vice versa).

Immune cell profiling was performed at various time points for diclofenac- and Tr14-treatments by analyzing changes in expression profiles of immune cell-type-specific marker genes. Figure 1C shows that macrophage, neutrophil, cytotoxic T cell, and natural killer cell markers increased immediately after treatment with diclofenac (time point 12 h). That was associated with upregulation of CD8^+^ T cell markers at 24 h, although the majority of the immune cell types were downregulated at later time points. In contrast to diclofenac, most immune cell type markers were upregulated in the Tr14 treatment group at 120 h. Of note, at this time point, the behavior of diclofenac and Tr14-treated immune cell markers demonstrated completely opposing effects suggesting that the largest difference between the treatments occurred at 120 h.

### Tr14 and diclofenac show opposite expression of cytokines and receptors in the late acute inflammatory response

**Fig. 2:**
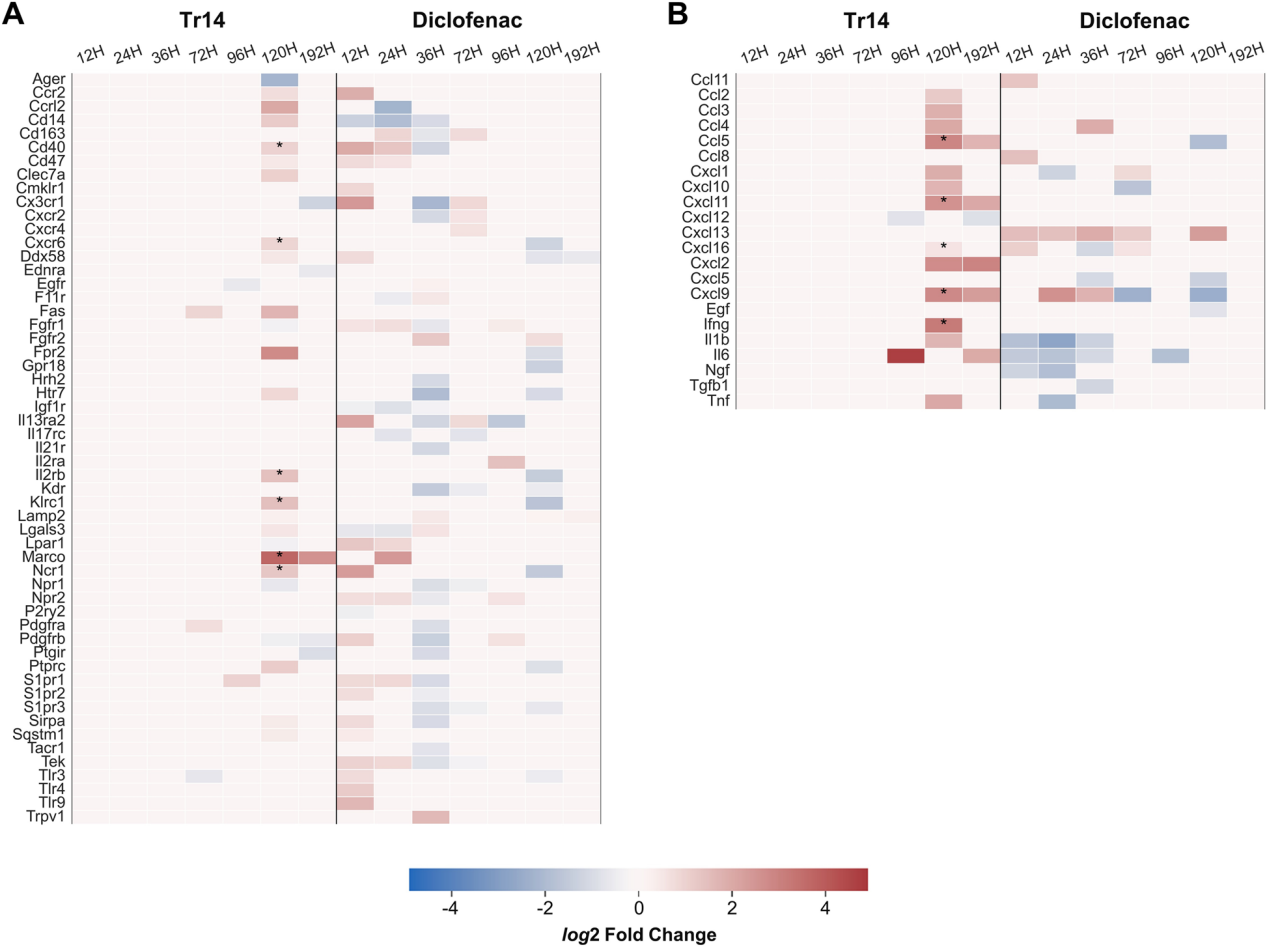
Selected receptor (**A**) and cytokine (**B**) genes associated with various immune cell types that were significantly differentially expressed at least at a one-time point in Tr14 and diclofenac treatment compared to their respective control. The heat map indicates that at time point 120 h, when most of the genes were upregulated by Tr14, they were downregulated by diclofenac. * Upregulated genes at 120 h with an increased base mean read count levels compared to all previous time points (12 h to 96 h)

The expression profiles of cytokines and receptor proteins present in the immune cell type-specific submaps on the AIR were identified in the two treatment groups compared to their respective controls (Fig. 2). Similar to the finding depicted in Fig. 1C, many of the related immune cell cytokines and receptors were significantly upregulated at time point 120 h in Tr14 while being downregulated at 36 h and 120 h in diclofenac treated animals. By comparison, at 12 h, most genes were upregulated by diclofenac treatment. Notably, Figs. 1C and 2 show independently that there is no overlap between the cell-type-specific genes and the cytokine or receptor DEGs. However, a significant differential expression refers only to a comparison of read count levels at the same time point and does not imply by default biologically relevant expression.

**Fig. 3:**
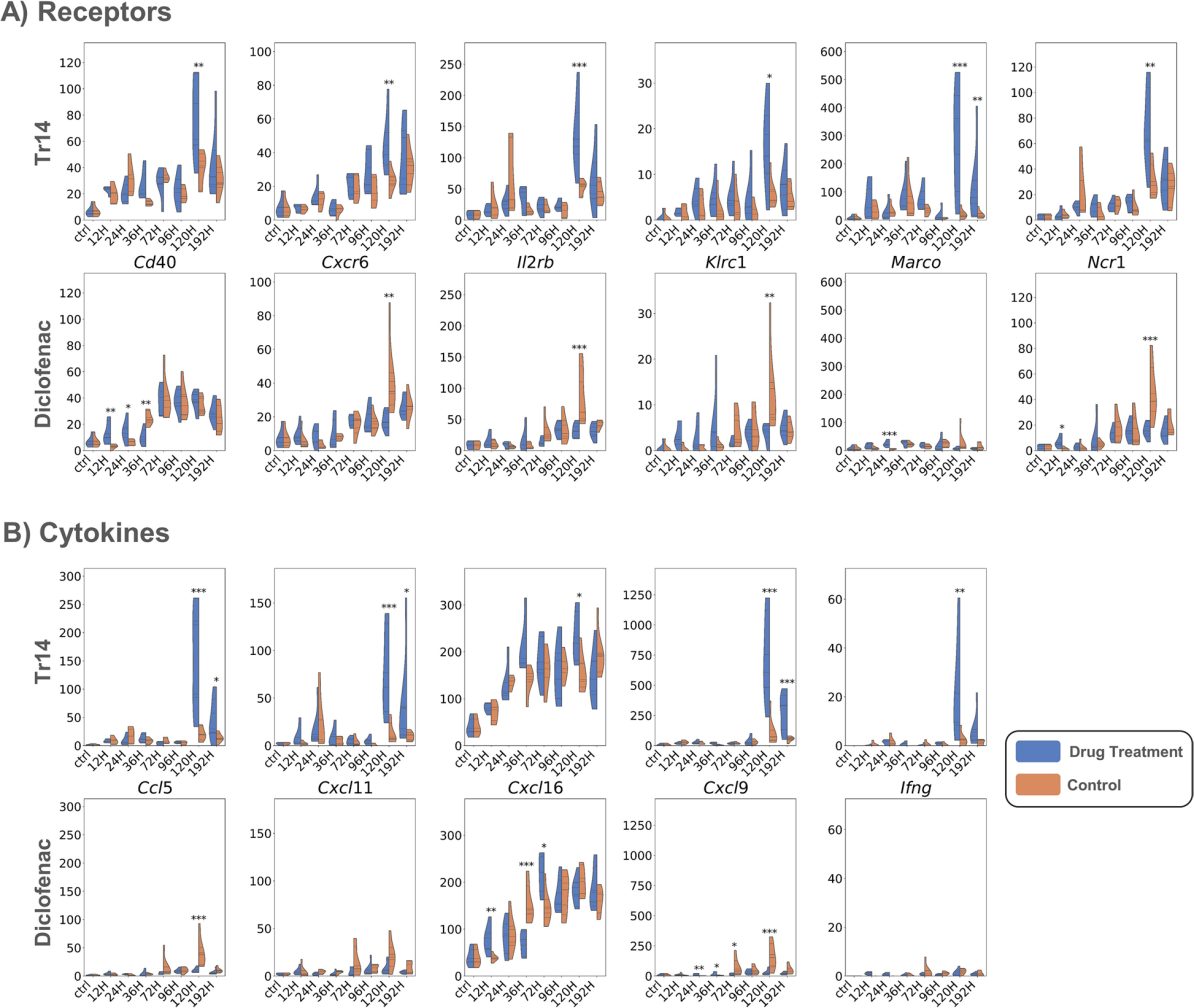
Read counts of receptors (**A**) and cytokines (**B**) present in the Atlas of Inflammation Resolution that were significantly induced in Tr14-treated samples (Tr14) compared to the saline control group. Violin plots showing the distribution of read counts in all samples for Tr14 vs. saline control and diclofenac vs. placebo control. * *p*-value < 0.05; ** *p*-value < 0.01; *** *p*-value < 0.001

Therefore, to identify genes with biologically relevant expression levels, we selected those with a higher base mean read count value at 120 h as compared to all earlier time points. Those genes are marked in Fig. 2 (*) and shown as separate violin plots in Fig. 3. For cytokines, the genes include *Ccl5*, *Cxcl11*, *Cxcl16*, *Cxcl9*, and *Ifng,* and for the receptors *Cd40*, *Cxcr6*, *Il2rb*, *Klrc1*, *Marco*, and *Ncr1*. Except for *Ifng*, all strongly induced cytokines are chemokines, further supporting an increased cellular response at 120 h in Tr14-treatment as shown by the increase in cellular marker genes in Fig. 1C. The plots in Fig. 3 again highlight the strong induction of these genes at 120 h in Tr14-treated samples, with elevated levels still visible at 192 h. In absolute and relative terms, the most remarkable increase at 120 h was observed for *Cxcl9*. As already shown in Fig. 2, a significant downregulation of the same genes can be seen at the same time point in the diclofenac treatment group.

**Fig. 4:**
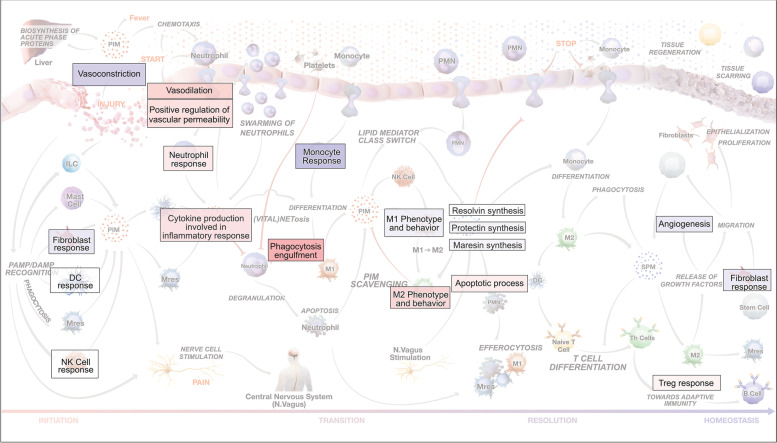
Predicted phenotype levels (red—> up, blue—> down) at 120 h after perturbation of DEGs with opposite expression profiles between the two treatment conditions. Using the Xplore plugin of the AIR, we perturbed those elements that were upregulated in Tr14 but downregulated in diclofenac-treated animals at 120 h (*Fpr2*, *Ddx58*, *Cxcr6*, *Klrc1*, *Il2rb*, *Ncr1*, *Htr7*, and *Ptprc*)

Of all the cytokines and receptor DEGs at 120 h, eight genes were subject to opposing regulation depending on whether the animal received Tr14 and diclofenac, namely *Fpr2*, *Ddx58*, *Cxcr6*, *Klrc1*, *Il2rb*, *Ncr1*, *Htr7*, and *Ptprc*. Of note, all of them were upregulated by Tr14. Their impact on inflammatory phenotypes on these genes was assessed by performing *in silico* perturbations on the AIR. Figure 4 shows the predicted changes in phenotype levels across the different stages of inflammation after upregulating all genes simultaneously in the Xplore tool. Processes that were positively affected by these perturbations included “apoptotic process,” “M2 phenotype and behavior,” and “apoptotic cell clearance,” which are consistent with the findings of Jordan et al. [30]. Together, these results suggest that Tr14 differs from diclofenac by impacting cellular and apoptotic processes in the later immune response, potentially optimizing wound clearing and repair.

### Tr14 and diclofenac have different modes of action on inflammatory phenotypes 

**Fig. 5:**
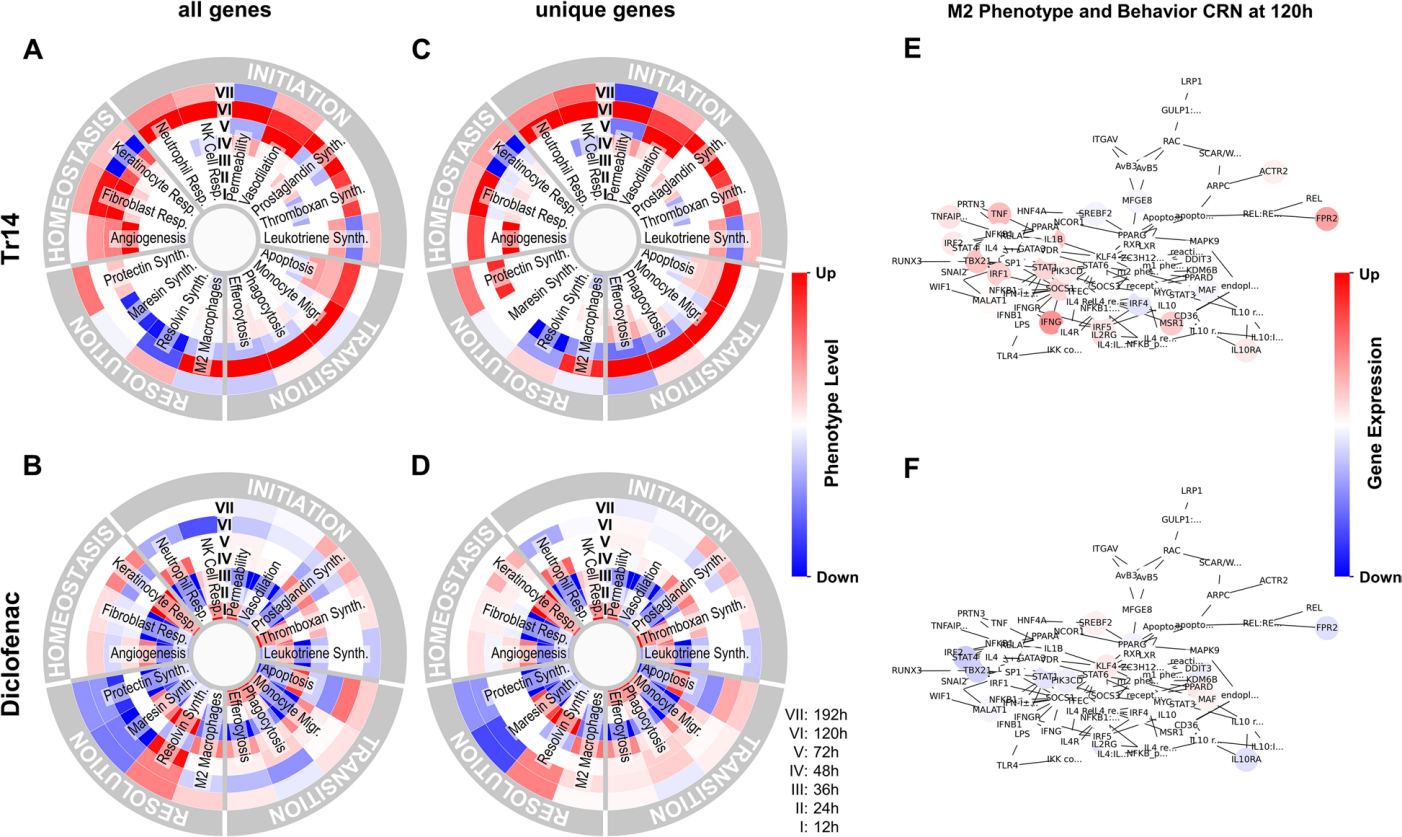
Impact on selected acute inflammatory processes and phenotypes in Tr14 vs. saline control (**A**, **C**, and **E**); and diclofenac vs. placebo control (**B**, **D**, and **F**). **A**-**D** The processes and phenotype levels were normalized between + 1 (upregulation; red color) and -1 (downregulation; blue color). Acute inflammatory processes and phenotypes were grouped into 4 phases (inflammation initiation, transition, resolution, and homeostasis). Circles from inner to outer regions represent treatment time points 12 h, 24 h, 36 h, 72 h, 96 h, 120 h, and 192 h. **E**–**F** For each process at a given time point and treatment, network- and expression-based motif ranking creates a central regulatory network (CRN) representing the molecular interaction associated with the selected phenotype element (e.g., M2 Phenotype and Behavior). The CRN highlights the up-regulated (red) or down-regulated (blue) differentially expressed genes (padj < 0.05) in the sample

In order to identify the mode of action differences for inflammatory phenotypes, we visualized predicted phenotype levels at different time points. Figure 5 shows selected upregulated (red) or downregulated (blue) processes/phenotypes at each time point during the four phases of acute inflammation described in the AIR, using either all DEGs or only the unique DEGs in each treatment condition (Fig. 1B). At early time points, most acute inflammation processes were downregulated in the diclofenac group compared to Tr14 treated mice. By comparison, at time point 120 h, many inflammation resolution processes were downregulated in the diclofenac treatment while being upregulated in the Tr14 treatment group; most of them were related to immune cell type activation. Treatment with Tr14 resulted in limited gene expression changes at 12 h and 24 h, but at 120 h, the effect peaked, especially on processes/phenotypes associated with acute inflammation resolution. Among diclofenac-treated mice, most of the selected acute inflammatory processes and phenotypes were affected at early time points compared with placebo-treated animals. In the diclofenac group, the highest number of significantly differentially regulated phenotypes occurred at 36 h. Interestingly, there were only small differences between the predicted phenotypes for both DEG sets indicating that phenotype enrichment was driven mainly by the unique DEGs. These findings further argue for a fundamental difference in the mode of action of both treatments.

### Phenotype-specific networks reveal differential effects of treatments on neutrophils and macrophages

To gain more insight into the molecular interactions underlying the predicted phenotypes, we identified phenotype-specific CRNs from the AIR MIM. When examining the CRNs for each phenotype, four processes, in particular, showed a substantial difference between the two treatments: “apoptotic process” (Figure S6), “NETosis” (Figure S7), “apoptotic cell clearence” (efferocytosis, Figure S8), and “M2 phenotype and behavior” (Figure S9). Whereas we observed downregulation of NETosis-inducing genes, such as *Padi4*, by Tr14 after 96 h, Tr14 also upregulated apoptosis-related genes (*Casp1*, *Casp3*, *Casp7*, and *Casp8*) and apoptosis-inducing receptors (*Fpr2*) after 120 h. At the same time point, Tr14 treatment resulted in a general upregulation of neutrophil marker genes (*Itgam*, *Ncf1*, and *Ncf2*). The expression of efferocytosis and M2 macrophage cytokine markers *Il4, Il10,* and *Il13* were too low to be detected in any of the treatments and time points. However, we see significant upregulation of many related receptor genes at 120 h by Tr14, including the *Il2rg* subunit of the IL4 receptor, the *Il10ra* subunit of the IL10 receptor, and the *Il13ra1* subunit of the IL13 receptor. An activation of efferocytosis by Tr14 would go hand in hand with its cytokine profile and the strong upregulation of phagocytotic markers. On the other site, diclofenac downregulated *Il2rg* and *Il10ra* at 120 h and the *Il4ra* subunit of the IL4 and IL13 receptor at 36 h. At 120 h diclofenac additionally downregulated *Fpr2* and upregulated *Padi4*. These results indicate that the neutrophil-macrophage axis is a central part in the different modes of action between Tr14 and diclofenac.

## Discussion

Inflammation is generally considered to be a protective immune response resulting in the elimination of damaged cells and pathogens. However, its timely resolution is essential to restoring tissue homeostasis [32–34]. The inflammatory response consists of multiple processes acting in a coordinated fashion. For this to happen, molecules exist which mediate the various phases, creating a tightly regulated system involving different tissues, cell types, cytokines, and receptors. Their impact on the acute inflammation spectrum largely depends on the phase of the inflammatory response [4], cell-type composition, tissue microenvironment [35, 36], including their crosstalk with the peripheral nervous system [37]. Conversely, there is no clear distinction between “friends” and “foes” in the molecular landscape of inflammatory processes as their roles can even be reversed in a spatiotemporal context [38, 39]. Subsequently, molecules that promote inflammatory phenotypes could indirectly affect various downstream processes associated with acute inflammation resolution. There are many molecular/phenotypic switches between various stages of acute inflammation that are regulated by feedback loops or alternative pathways. For example, switching from M1 to M2 macrophages is regulated by a double feedback loop between miR-23a/27a/24–2 [40], a negative feedback loop between CKIP-1 and M1 and M2 cytokines [41], and a positive feedback loop between MaR1, RvD2 and M2 macrophages [42]. Similarly, the switching from pro-inflammatory lipid mediators (PIM) to specialized pro-resolving lipid mediators (SPM) is regulated through a negative feedback loop between 15dPGJ2 and COX-2 [43] and a negative feedback loop between PGE2, DUSP1, TTP, and COX-2 [44]. The submaps available on the AIR related to the biosynthesis of PIM and SPM also highlight several alternative pathways from various precursor lipid molecules and metabolic enzymes [4].

In this study, the effects of a multicomponent drug Tr14 on cell types and processes associated with wound healing and differential gene expression were investigated and compared with those for diclofenac. Apart from unique differentially expressed genes at various time points in both treatment groups, we found that more than 40% of the common DEGs were expressed in opposite directions (upregulated in Tr14 but downregulated in the diclofenac group compared to their respective controls or vice versa). The heatmap of unique DEGs (Fig. 1A) also shows that the responses to treatment with diclofenac are very intense and immediate, while Tr14 results in large numbers of DEGs appearing from 72 h onward. These findings suggest that the effects of treatments differ in timing during acute inflammation. Correspondingly, the results of our phenotype enrichment analysis showed that Tr14, although having minimal effects at early time points, strongly induces cellular responses associated with the resolution of inflammation 72 h after induction in a murine wound model. By comparison, diclofenac strongly modulates anti-inflammatory processes immediately after the start of treatment (Fig. 2) and negatively impacts the late cellular response. These results suggest that Tr14 has an advantage in upregulating key processes associated with inflammation resolution followed by homeostasis. The in vivo evidence about the pro-resolution properties of Tr14 was previously highlighted in the transcriptomics analysis of wound healing mouse model [24, 27]. The authors suggested that the pro-resolution properties of Tr14 could be attributed to the lack of COX2 inhibition in contrast to diclofenac. Another study by Jordon and colleagues [30] highlighted the pro-resolution effects of Tr14 in a well-establish model of mouse zymosan-induced peritonitis, which showed a shortening of resolution interval, suggesting an accelerated resolution by Tr14. Although the authors did not compare Tr14 with diclofenac, they further highlighted accelerated Tr14-mediated resolution in human macrophages in the same study. In previous clinical studies in humans, Tr14 showed non-inferior to diclofenac in reducing pain and improving mobility after injury [31, 45]. Although human studies did not directly measure the inflammation resolution or the wound healing properties of Tr14, the clinical outcomes indirectly support animal studies' findings.

Consistent with our results, Laurent et al*.* found that angiogenesis is one of the major processes upregulated by Tr14 at 120 h [24]. Using GO-enrichment analysis, St. Laurent and colleagues found similar processes to be enriched by Tr14, such as “Leukocyte Migration,” “Chemokine Activity,” “Cell Proliferation,” or “Apoptotic Process” [27]. The interpretation of transcriptome data has proven difficult, as thousands of genes need to be scanned, and thus interrelationships can be quickly missed. Using the AIR molecular interaction network and our recently developed network-based enrichment approach, we overcame these challenges by automatically identifying differentially expressed marker genes directly linked to predicted processes and phenotypes. Complementing the work of Laurent et al. [24, 27], our analyses revealed causal interactions behind the phenotype estimates of both studies and provided new insights into the mode of action of both treatments.

The major differences between Tr14 and diclofenac revealed by the phenotype-specific networks of the AIR can be divided into four groups:

(I) Genes of cellular functions: From the CRNs related to neutrophil and macrophage function, we assume that the downregulation of proinflammatory NETosis genes by Tr14 results in longer survival of neutrophils, and thus an increase of neutrophil marker genes and induction of neutrophil apoptosis. The following phagocytosis of apoptotic neutrophils by macrophages, called efferocytosis, is a critical step toward inflammation resolution and has been shown to stimulate tissue cleansing and repair [46, 47]. This hypothesis is supported by the increased expression of Il4-, Il10-, and Il13-receptor genes by Tr14. In diclofenac, most of these genes were oppositely regulated. We also found a higher activity of glycolysis through increased expression of hexokinase and phosphofructokinase genes in Tr14-treated samples at 120 h. In the CRNs these enzymes are connected to HIF-1α, which is upregulated by Tr14 at 120 h as well and plays an important role in immune function through stimulation of glycolysis [48].

In a separate study on a zymosan-induced mouse peritonitis model, Jordan and colleagues found a significantly higher level of SPMs at time point 24 h when mice were injected with 3 ml/kg body weight of Tr14 through *i.p.* and at a late time point (360 h) with a low concentration of Tr14 (1.5 ml/kg *i.p.*) [30]. In the current analysis, we observed an upregulation of SPM biosynthesis, specifically for resolvins and protectins, at the late time point (192 h) in Tr14 treatment and very early time point (24 h) in diclofenac treatment on resolving biosynthesis (Fig. 5). In the peritonditis model, Jordan and colleagues also observed in increase in efferocytotic macrophages at very early time points (4 h). Moreover, their results suggest that the application of Tr14 increases SPM synthesis by M2 macrophages in cell culture. Similarly, our network analysis revealed upregulation of gene interactions related to efferocytosis and M2 macrophage. The independent and consistent predictions of both studies support an effect of Tr14 on macrophage efferocytosis, M2 polarization, and SPM synthesis.

(II) Non-chemotactic cytokines: IL6 expression was either downregulated or statistically not significant in the diclofenac treatment group compared to topical placebo control, while it was highly upregulated (log2FC > 4) in the Tr14 treatment group at time point 96 h compared to saline control (Figure S2). IL6 signaling represents a crucial checkpoint for neutrophil trafficking, chemokine production, leukocyte apoptosis, and thus the termination of the innate immune response [49]. Another cytokine, IFNG, is also upregulated by Tr14 at 120 h. IFNG has been shown to be required for proper wound closure, especially, consistent with our other findings, in the proliferative phase of the wound healing [50]. In Tr14-treated samples, we also observed a significant increase in TNF and IL1B at 120 h. However, their read counts in both the treatment and control at 120 h are much lower than in all the earlier time points, making it questionable whether the increase is due to induction or simply a higher number of expressing cells.
(III) Chemokines: CXCR9 and CXCL11, both CXCR3 receptor ligands and strongly upregulated by Tr14 at 120 h, are essential chemokines in wound healing processes [51, 52]. For CCL5 and CCRL2, involvement in the late immune response, T-cell activation, pathways associated with the resolution of inflammation [53, 54] and in wound healing [55] has been reported. Other elements of the CCL5 axis, such as CCL9, NOS2, TNF, and IL1B [56, 57] are also upregulated in Tr14 at 120 h and promote stem cell invasion and wound healing through cellular processes [58]. Diclofenac on the other site strongly downregulated CXC9 and CCL5. These findings support a more substantial effect of Tr14 on the cellular immune response, especially affecting the adaptive immune system, thus promoting long-lasting tissue clearance, and stimulating regenerative processes.
(IV) Cell type markers and receptors: In the case of Tr14 treatment, we observed high expression of FPR2 at later treatment time points, while it was downregulated following administration of diclofenac. Many specialized proresolving mediators, including LXA4 and RvD1, bind with ALX/FPR2 receptors which are central to the resolution of the inflammation [59, 60]. Two genes that occurred in many CRNs were IL2RB and IL2RG, both subunits of the IL2 receptor which is expressed on many cell types during the proliferative phase [61]. Our cell type composition analysis using the differential expression of cell-type-specific genes adds weight to these findings and the chemokine profiles. Laurent et al. also reported increases in another set of cell-type markers related to the adaptive immunity [27]. Collectively, the available information point to Tr14 being able to induce many immune cell types and processes at 120 h. By comparison, diclofenac initially upregulated some cell types while suppressing most immune cell types and processes, especially at 36 h and 120 h.

Cell type analysis from bulk RNA-sequence data is limited by shifts in cell compositions between samples, affecting the quantity of transcript reads available, leading to false assumptions on transcriptional differences [62]. In the present study, we countered these limitations by including and averaging only uniquely highly expressed genes in respective cell types. The network-based enrichment analysis of the AIR facilitated the identification of genes with high relevance to each process. The network does not include mechanistic information but allows for statistical assessment of overrepresented phenotypes. While it is possible to estimate whether a process may have been more or less active at a specific time point, it is not possible comparing the activities between different processes and, therefore, should be avoided. The three day-difference between the 120 h and 192 h time points limit the interpretability of the inflammatory processes regulated during that time.

In summary, we found that Tr14 induced opposite transcriptional changes compared to diclofenac, especially at 120 h. Conversely, some processes induced by Tr14 at 120 h are also induced by diclofenac, although already at 36 h. One explanation may be that the early inhibitory effect of diclofenac on inflammation causes some processes to shift in their timely activation while others remain blocked. Our observations suggest that Tr14 strengthens the late physiological immune response otherwise downregulated at an earlier stage by the anti-inflammatory drug diclofenac. The difference in the phenotypic effects of the two treatments may have been caused by their fundamentally different pharmacodynamic nature. Diclofenac, as an NSAID, has a direct, potent inhibitory effect on cyclooxygenase enzymes (PTGS1 and PTGS2), leading to noticeable changes in downstream signaling and metabolic cascades associated with SPM biosynthesis [27, 63, 64]. Following administration, an initial effect on early gene transcription continued over time. By comparison, the multicomponent drug Tr14 appears initially to have a lesser effect. On the contrary, Tr14, as a multicomponent natural product, presumably modulates the SPM biosynthesis or its effects through multitarget mechanisms. Consequently, the early effect of Tr14 on the lipid mediator pathway on individual targets might not be directly detectable at the transcriptional level, especially in bulk tissue samples.

It may seem counterintuitive that Tr14 is positioned here as an anti-inflammatory and pro-resolution drug even though it increased gene expression for many pro-inflammatory genes, in contrast to the suppression of many of these same genes by diclofenac. However, accumulating evidence suggests that a pro-inflammatory phenotype at the early stages of acute inflammation is an essential requirement to promote inflammation resolution and restore tissue homeostasis [65]. Early and immediate suppression of pro-inflammatory signals shown to have various long-term chronic complications. For example, Parisiens and colleagues in the rodent model of acute pain showed that after an acute lower back injury, the active suppression of neutrophil influx using NSAIDs or corticosteroids might provide short-term analgesia but lead to prolonged pain [66]. Similarly, in a rat model of Achilles tendon rupture, dexamethasone improves tendon healing and restores functionality compared to placebo only when injected after, but not during, the early inflammatory phase [67]. These results suggest that events occurring during the early acute inflammatory phase are needed for tissue healing. During the time course of acute inflammation, certain inflammatory cells, including neutrophils and macrophages, undergo functional repolarization to acquire phenotypes that contribute to the onset of inflammation resolution. Additionally, some mediators that initially promote the proinflammatory phase, including PGE-2, can switch roles to initiate a program for active resolution [44]. Whether different mediators act in a proinflammatory, anti-inflammatory, or pro-resolution manner is determined in part by their spatiotemporal relationships with other cells and the surrounding microenvironment during the entire time course of acute inflammation.

Assuming that Tr14 acts simultaneously and slowly on multiple molecular targets, we hypothesize that small changes in regulatory components accumulate over time and lead to significant late modulation of the inflammatory response without disrupting important initial processes [68–70]. We suggest that the use of multitarget drugs with smaller but longer-lasting influences on different cellular processes could be of greater clinical value in reducing inflammation and improving inflammation resolution over time, compared to drugs with a strong, early inhibitory effect.

## Methods

### Data acquisition

The data analyzed in this data has been published previously [24, 27]. Laurent and colleagues reported the effects of Tr14 and diclofenac treatment on gene expression in a murine model of a surgical skin wound of 1 cm^2^ abraded with rotary abrasive tool. Injured mice were treated daily with Tr14 (injection + topical), diclofenac (topical), or a placebo (control: saline injection and/or topical, respectively). At times 0, 12, 24, 36, 72, 96, 120, and 192 h after surgical incision, seven mice from each treatment group were sacrificed, tissue samples obtained, and gene expression analyzed using RNA-sequencing. For our analysis we downloaded the preprocessed data containing read count values of prealigned transcripts from https://trace.ncbi.nlm.nih.gov/Traces/study/?acc=PRJNA726431 [27].

### Data processing

Read counts were filtered, selecting transcripts with the maximum exon length for each gene. Using the DeSeq2 R package, read counts for all samples were normalized based on sample library size. The normalized read counts for each sample and time point were examined using principal component analysis (PCA) to identify outliers that might affect the statistical analysis. After removing the outliers from the raw data, the time-series transcriptomics data was analyzed using the DESeq2 R package. *Log*2 fold change values (FC) and adjusted *p*-values (*p*_adj_) were calculated for the Tr14 and diclofenac treatment groups compared to their respective placebo controls at seven different time points starting from 12 to 192 h, respectively (Additional file 1). We then selected differentially expressed genes (DEGs) with *p*_adj_ < 0.05. All the DEGs were mapped to the molecular interaction map (MIM) associated with the AIR.

### Systems biology approach and the Atlas of Inflammation Resolution (AIR)

**Fig. 6:**
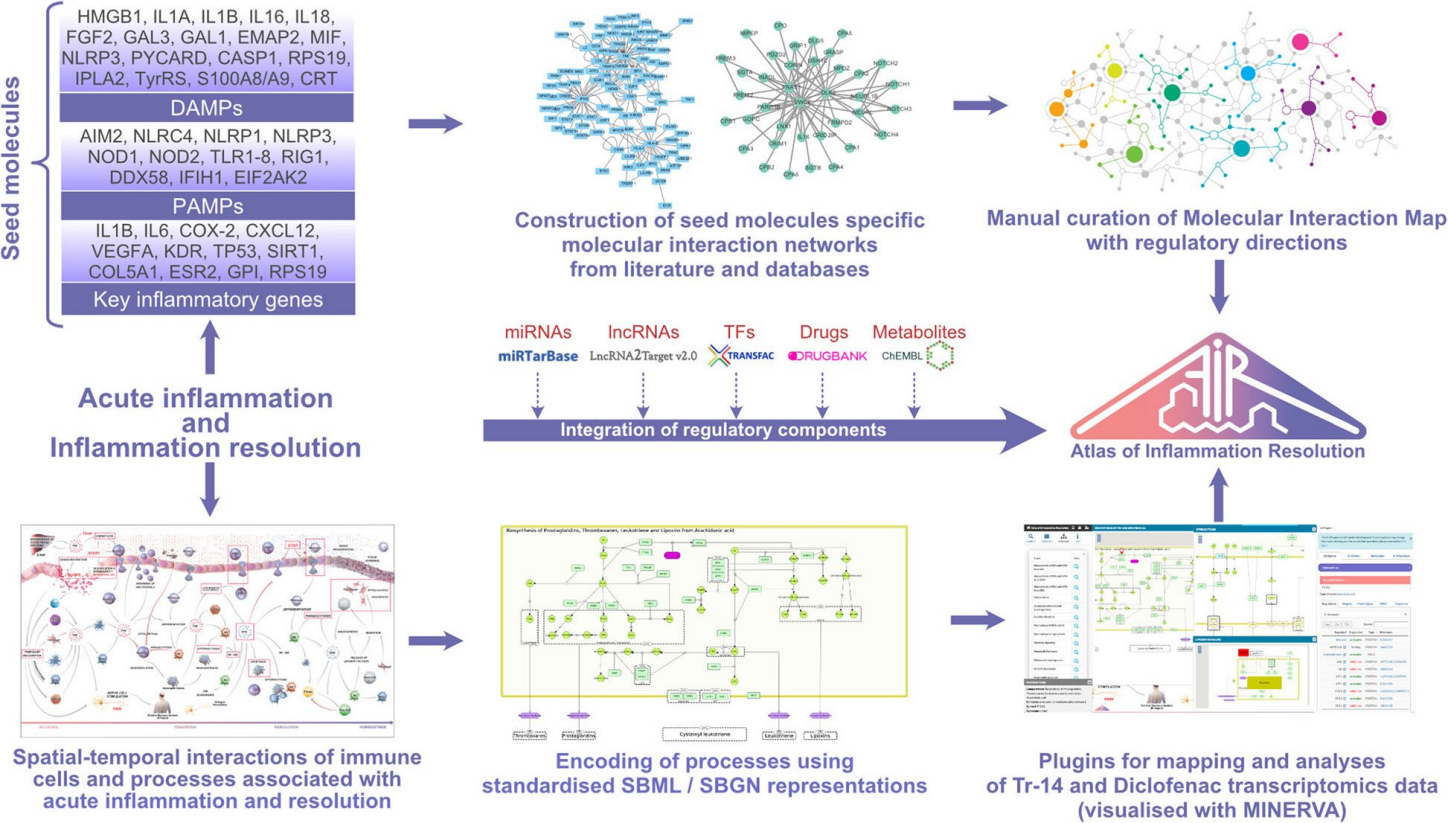
Workflow employed to construct a comprehensive molecular interaction map to examine acute inflammation. The process began with the identification of seed molecules (left panel). This was followed by literature mining and the extraction of experimentally validated interacting molecular partners using several databases (middle). The final step (right) was to integrate experimentally validated regulatory layers of transcription factors, miRNA, and long non-coding RNAs from state-of-the-art databases. Various metabolites associated with acute inflammation and inflammation resolution were included together with their biosynthesis and signaling pathways. These maps are publicly available at https://air.bio.informatik.uni-rostock.de

We first expanded the knowledge base of the AIR by adding new information about molecular and signaling activity associated with acute inflammation resolution. This included information on the role of macrophage polarization, signaling cascades of various immune cells, such as T- and B-cells. The workflow for the construction of the AIR is summarized in Fig. 6. Next, the AIR was used to analyze the RNA-Sequence time-series data for diclofenac and Tr14 treatment responses in the wound healing model [27].

Using a systems biology approach for the AIR, we developed two plugins (Omics and Xplore) comprising various tools to integrate and analyze multi-omics data and explore the role of feedback mechanisms in molecular interaction networks [71]. The tools enable *in silico* perturbations and network-based enrichment experiments to identify regulated immunological phenotypes and processes.

### Phenotype enrichment analysis

**Fig. 7:**
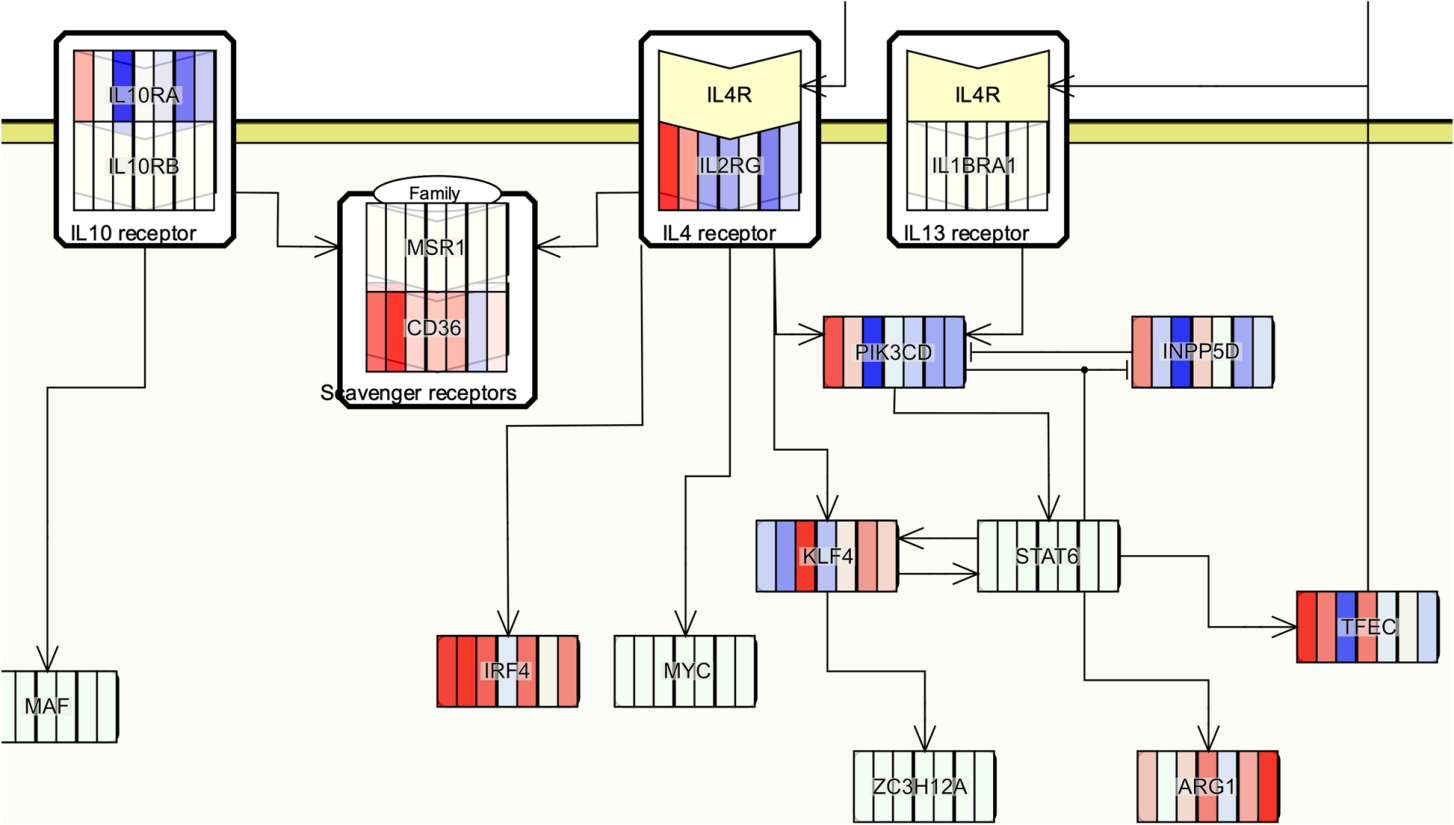
Mapping time-series transcriptomics data onto the Atlas of Inflammation Resolution to estimate downstream processes and phenotypes. Each bar represents a log2 fold change (red: up; blue: down) of an individual gene’s read count at a given time point

The FC and *p*_adj_ for DEGs were compiled in two tab-separated text files for Tr14 vs. saline control and diclofenac vs. placebo control samples, respectively. The information included official gene symbols as a single column and separate columns for FC and *p*_adj_ values for each time point. The file contents were then integrated within the AIR using the ‘Omics’ analysis plugin (Fig. 7). Using the phenotype enrichment tool of the plugin, we identified significantly differentially regulated phenotypes to determine the effects of Tr14 and diclofenac on different acute inflammatory processes. Further, we ranked the regulators of each phenotype by their expression value and influence score. The methodology underlying the plugin is described in detail in our recent work [71]. The analysis was complemented by a literature search for additional evidence on the highest-ranked regulators in the context of acute inflammation and resolution.

### In silico perturbation analysis

Cytokines and cytokine receptors from all the DEGs included in the AIR were studied in greater detail. DEGs upregulated/downregulated in both Tr14 and diclofenac groups were identified. The cytokines and receptor genes with opposite expression regulation after respective treatments (e.g., positive regulation in one group, but negative in another) were selected for *in silico* perturbation experiments using the Xplore plugin. Based on the expression patterns for selected genes, values were designated as either + 1 or -1, and their impact on inflammatory phenotypes was observed.

### Cell type composition analysis

Cell-gene annotations from the ‘nCounter® Mouse PanCancer Immune Profiling Panel’ were acquired. The results, comprised of 93 marker genes, were grouped into 13 different immune cell types (available on https://www.nanostring.com). Marker genes representing significant DEGs in the dataset (*p*_adj_ < 0.05) were identified for each treatment time point. The expression profile was then averaged to estimate the log2 fold-change in immune cell composition. Cell types with opposing marker gene FC values were ignored in the cell type composition analysis.

### Core regulatory network identification

To generate subnetworks underlying the phenotype predictions, we adapted the work from Khan et al. in 2017 [72]. The approach is based on the ranking of motifs, which are gene triplet feedback loops, by scoring and weighted normalized network and expression features. By iteratively changing the weights and selecting the highest ranked motifs at each iteration, a Pareto set of motifs with the highest feature values is generated. These motifs are then merged into a single network, a so-called core regulatory network (CRN). We adapted the algorithm to include features generated by the phenotype prediction algorithm. For a selected phenotype $p$, the score for a $k$-mer motif with a weighting scenario $j$ is calculated as shown in Eq. 1 with $\langle I\rangle$: influence score on p; $\langle ES\rangle$: upstream enrichment score; $\langle \left|FC\right|\rangle$: absolute *log*2 fold change value; and $\left\{{w}_{1};{w}_{2};{w}_{3}\right\}\subseteq \{0.33;0.66;1.0\}$. In addition, we integrated the functionality for creating interactive CRNs into the user interface of the AIR analysis tools (https://air.bio.informatik.uni-rostock.de/plugins).

1 $${S}_{j}={w}_{1j}\cdot \sum_{i=1}^{k}{I}_{i,p}+{w}_{2j}\cdot \sum_{i=1}^{k}{ES}_{i}+{w}_{3j}\cdot \sum_{i=1}^{k}{\left|FC\right|}_{i}$$

## Conclusions

We investigated whether an immunomodulatory drug that acts simultaneously at multiple sites (Tr14) shows greater potential to resolve acute inflammation more effectively and with fewer side effects than a common anti-inflammatory drug developed as a small molecule for a single target (diclofenac). Using our previously published Atlas of Inflammation Resolution, we examined altered gene expression associated with inflammatory processes and cellular profiles. The timely and effective transition from the proinflammatory phase to the pro-resolution phase of acute inflammation, including the synthesis of pro-resolving mediators and the development of an M2 phenotype, relies on certain cellular and molecular mechanisms that occur early in the time course [73]. Tr14, unlike diclofenac, does not suppress the expression of proinflammatory genes earlier in the time course of acute inflammation but supports the expression of genes later in the time course, suggesting it meets the essential criterion of a pro-resolution drug. Comparing the two treatments, we found both opposing responses and temporal differences, suggesting markedly different pharmacodynamics of multitarget and single-target drugs in resolving inflammation. Our results provide new insights into the molecular and cellular mode of action of both treatments in acute inflammation.

## Supplementary Information

**Additional file 1: Figure S1.** PCA analysis of RNA-seq samples for Tr14 treatment (A) and saline control (B) at 12h. Outliers (orange) were removed afterward. (C and D) Volcano plots showing the impact of outlier removal on the DeSeq2 differential analysis for Tr14 vs. control. (E) Venn diagram comparing the number of significant genes (adj. *p*-value < 0.05) before and after the removal of outliers. **Figure S2.** PCA analysis of RNA-seq samples for Tr14 treatment (A) and saline control (B) at 72h. Outliers (orange) were removed afterward. (C and D) Volcano plots showing the impact of outlier removal on the DeSeq2 differential analysis for Tr14 vs. control. (E) Venn diagram comparing the number of significant genes (adj. *p*-value < 0.05) before and after the removal of outliers. **Figure S3.** PCA analysis of RNA-seq samples for Tr14 treatment (A) and saline control (B) at 96h. Outliers (orange) were removed afterward. (C and D) Volcano plots showing the impact of outlier removal on the DeSeq2 differential analysis for Tr14 vs. control. (E) Venn diagram comparing the number of significant genes (adj. *p*-value < 0.05) before and after the removal of outliers. **Figure S4.** PCA analysis of RNA-seq samples for Tr14 treatment (A) and saline control (B) at 120h. Outliers (orange) were removed afterward. (C and D) Volcano plots showing the impact of outlier removal on the DeSeq2 differential analysis for Tr14 vs. control. (E) Venn diagram comparing the number of significant genes (adj. *p*-value < 0.05) before and after the removal of outliers. **Figure S5.** PCA analysis of RNA-seq samples for diclofenac treatment (A) and placebo control (B) at 96h. Outliers (orange) were removed afterward. (C and D) Volcano plots showing the impact of outlier removal on the DeSeq2 differential analysis for diclofenac vs. control. (E) Venn diagram comparing the number of significant genes (adj. *p*-value < 0.05) before and after the removal of outliers. **Figure S6.** Core Regulatory Networks (CRNs) of the “apoptotic process” phenotype for Tr14 and Diclofenac treatment at 96 and 120 hours. Gene triplets in the molecular interaction map connected to the phenotype are ranked by *log*2 fold change values and network features. The highest ranked motifs were than selected and merged into the CRNs. **Figure S7.** Core Regulatory Networks (CRNs) of the “NETosis” phenotype for Tr14 and Diclofenac treatment at 96 and 120 hours. Gene triplets in the molecular interaction map connected to the phenotype are ranked by *log*2 fold change values and network features. The highest ranked motifs were than selected and merged into the CRNs. **Figure S8.** Core Regulatory Networks (CRNs) of the “apoptotic cell clearance” (efferocytosis) phenotype for Tr14 and Diclofenac treatment at 96 and 120 hours. Gene triplets in the molecular interaction map connected to the phenotype are ranked by *log*2 fold change values and network features. The highest ranked motifs were than selected and merged into the CRNs. **Figure S9.** Core Regulatory Networks (CRNs) of the “M2 phenotype and behavior” phenotype for Tr14 and Diclofenac treatment at 96 and 120 hours. Gene triplets in the molecular interaction map connected to the phenotype are ranked by *log*2 fold change values and network features. The highest ranked motifs were than selected and merged into the CRNs.

**Additional file 2: **Excel sheets containing adjusted p-values and log2 fold change values from the DESeq2 analysis of Tr14 and Diclofenac RNAseq data compared to their respective controls at timepoint 12h, 24h, 36h, 48h, 72h, 120h, and 192h.

**Additional file 3: **List of unique significantly differentially expressed genes (adjusted p-value < 0.05) in the Tr14 and Diclofenac treatment groups compared to their respective controls across all the time points.

## Data Availability

Read count values of preprocessed and prealigned transcripts, including each animal at each time point in every group, are available at https://trace.ncbi.nlm.nih.gov/Traces/study/?acc=PRJNA726431. Results of the DESeq2 analyses, containing fold change values and adjusted *p*-values for all genes at each time point in every group, are submitted for public access in Additional file 2.

## Abbreviations

AIR: Atlas of Inflammation Resolution
COX-1: Cyclooxygenase 1
COX-2: Cyclooxygenase 2
DEGs: Differentially expressed genes
FC: *log*2 Fold change
MIM: Molecular interaction map
NSAID: Non-steroidal anti-inflammatory drug
*p*_adj_: Adjusted *p*-value
PCA: Principal component analysis
PGE2: Prostaglandin-E2
PIM: Pro-inflammatory lipid mediators
SPMs: Specialized pro-resolving lipid mediators
Tr14: Traumeel
