# Supplementary material for: Network analyses reveal new insights into the effect of multicomponent Tr14 compared to single-component diclofenac in an acute inflammation model
Source: J Inflamm (Lond). 2023 Mar 27;20:12. doi: 10.1186/s12950-023-00335-0 (PMC10044762; doi:10.1186/s12950-023-00335-0)
Supplement: Supplementary file 1 — Additional file 1: Figure S1. PCA analysis of RNA-seq samples for Tr14 treatment (A) and saline control (B) at 12h. Outliers (orange) were removed afterward. (C and D) Volcano plots showing the impact of outlier removal on the DeSeq2 differential analysis for Tr14 vs. control. (E) Venn diagram comparing the number of significant genes (adj. p-value < 0.05) before and after the removal of outliers. Figure S2. PCA analysis of RNA-seq samples for Tr14 treatment (A) and saline control (B) at 72h. Outliers (orange) were removed afterward. (C and D) Volcano plots showing the impact of outlier removal on the DeSeq2 differential analysis for Tr14 vs. control. (E) Venn diagram comparing the number of significant genes (adj. p-value < 0.05) before and after the removal of outliers. Figure S3. PCA analysis of RNA-seq samples for Tr14 treatment (A) and saline control (B) at 96h. Outliers (orange) were removed afterward. (C and D) Volcano plots showing the impact of outlier removal on the DeSeq2 differential analysis for Tr14 vs. control. (E) Venn diagram comparing the number of significant genes (adj. p-value < 0.05) before and after the removal of outliers. Figure S4. PCA analysis of RNA-seq samples for Tr14 treatment (A) and saline control (B) at 120h. Outliers (orange) were removed afterward. (C and D) Volcano plots showing the impact of outlier removal on the DeSeq2 differential analysis for Tr14 vs. control. (E) Venn diagram comparing the number of significant genes (adj. p-value < 0.05) before and after the removal of outliers. Figure S5. PCA analysis of RNA-seq samples for diclofenac treatment (A) and placebo control (B) at 96h. Outliers (orange) were removed afterward. (C and D) Volcano plots showing the impact of outlier removal on the DeSeq2 differential analysis for diclofenac vs. control. (E) Venn diagram comparing the number of significant genes (adj. p-value < 0.05) before and after the removal of outliers. Figure S6. Core Regulatory Networks (CRNs) of the “apopto [file 12950_2023_335_MOESM1_ESM.pdf]

Tr14 vs. saline control at 12h

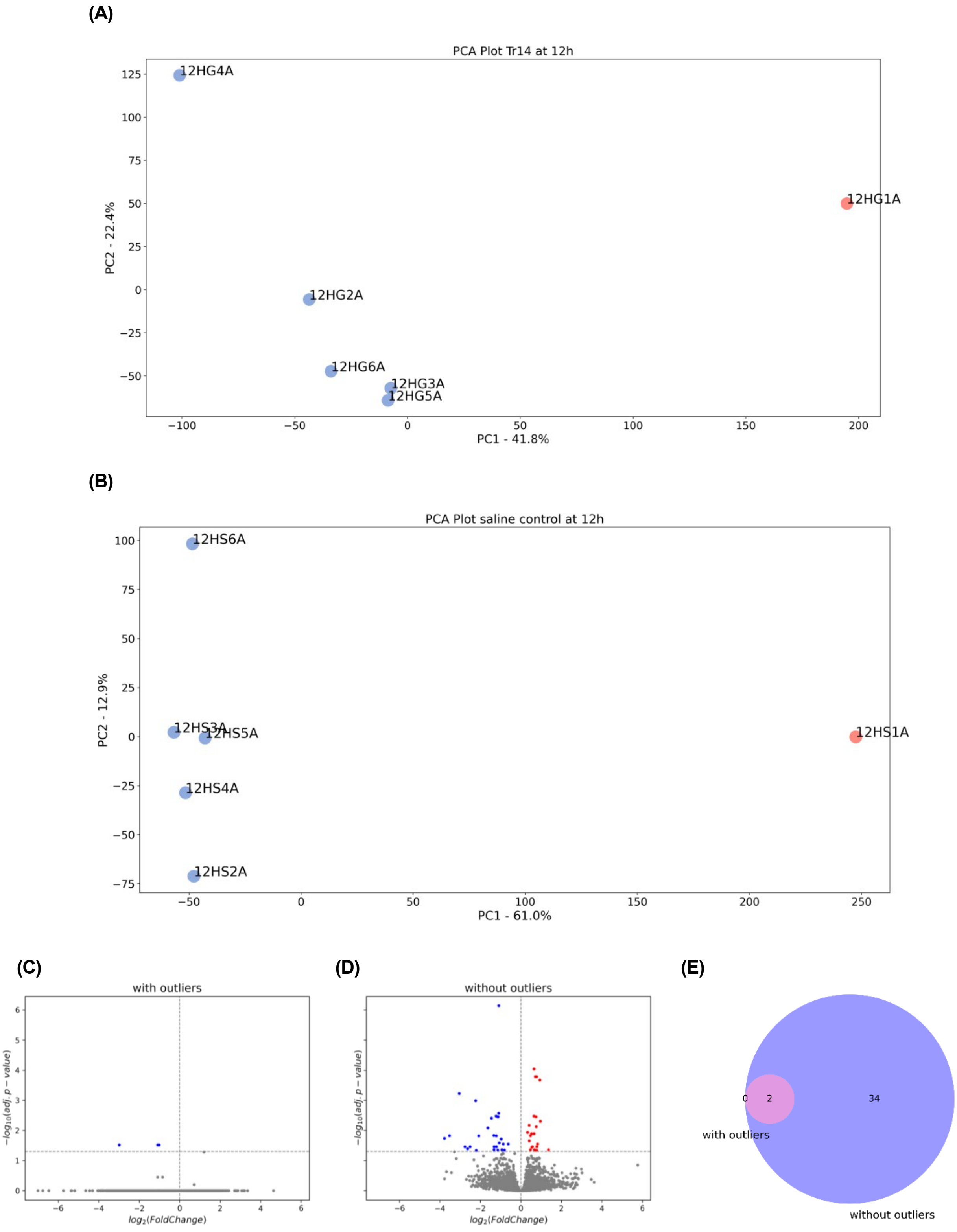

Figure S1: PCA analysis of RNA-seq samples for Tr14 treatment (A) and saline control (B) at 12h. Outliers (orange) were removed afterward. (C and D) Volcano plots showing the impact of outlier removal on the DeSeq2 differential analysis for Tr14 vs. control. (E) Venn diagram comparing the number of significant genes (adj. *p*-value < 0.05) before and after the removal of outliers.

Tr14 vs. saline control at 72h

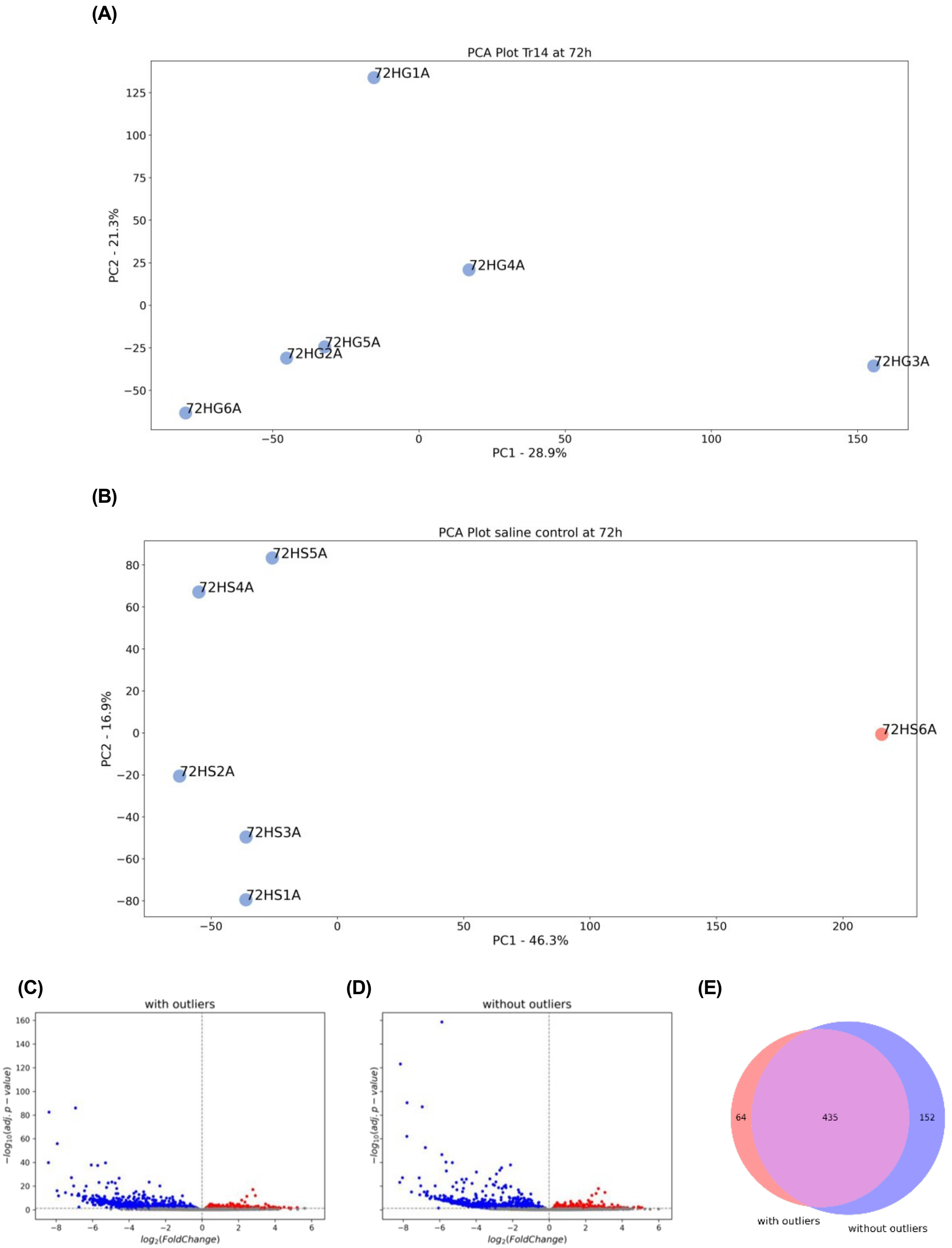

Figure S2: PCA analysis of RNA-seq samples for Tr14 treatment (A) and saline control (B) at 72h. Outliers (orange) were removed afterward. (C and D) Volcano plots showing the impact of outlier removal on the DeSeq2 differential analysis for Tr14 vs. control. (E) Venn diagram comparing the number of significant genes (adj. *p*-value < 0.05) before and after the removal of outliers.

Tr14 vs. saline control at 96h

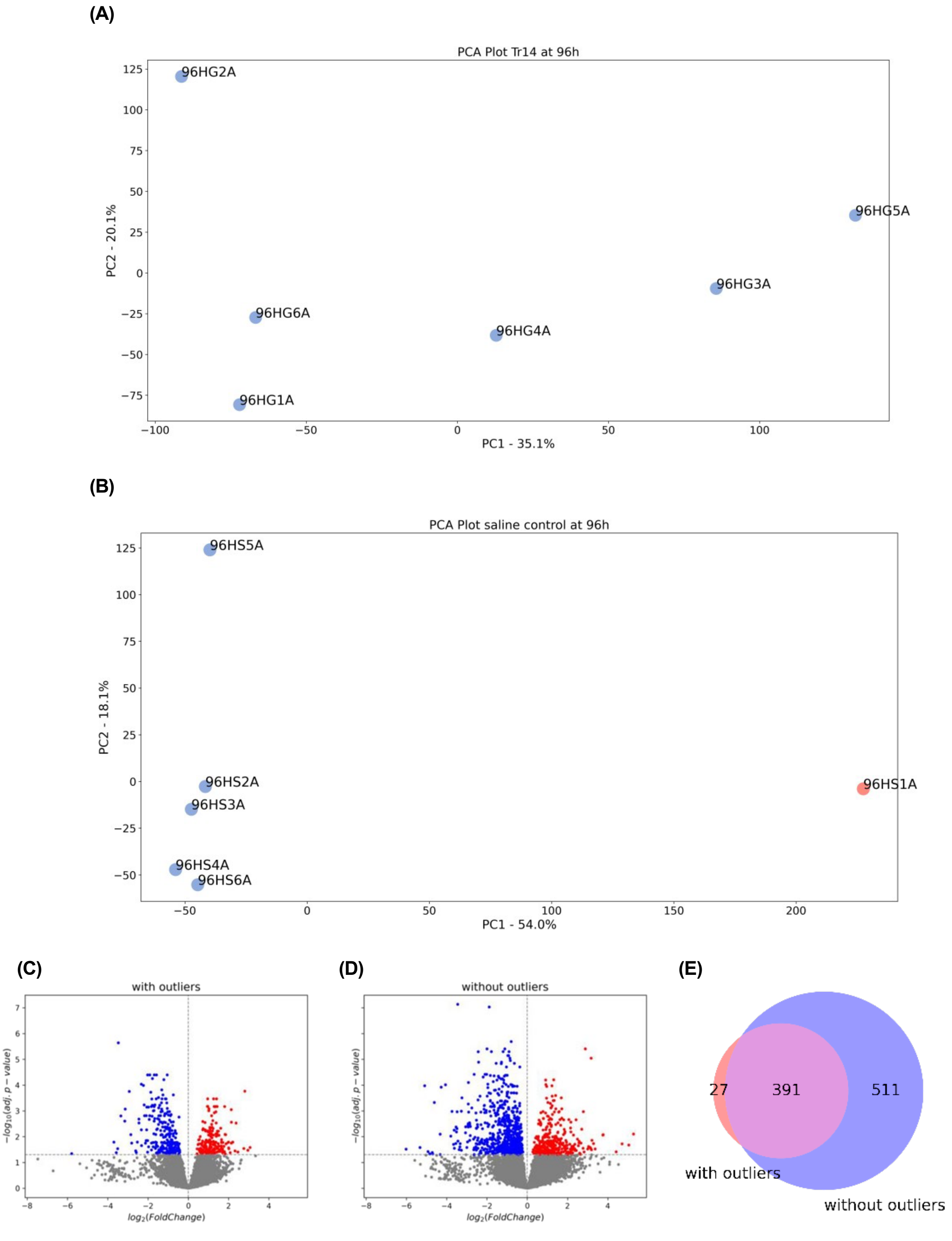

Figure S3: PCA analysis of RNA-seq samples for Tr14 treatment (A) and saline control (B) at 96h. Outliers (orange) were removed afterward. (C and D) Volcano plots showing the impact of outlier removal on the DeSeq2 differential analysis for Tr14 vs. control. (E) Venn diagram comparing the number of significant genes (adj. *p*-value < 0.05) before and after the removal of outliers.

Tr14 vs. saline control at 120h

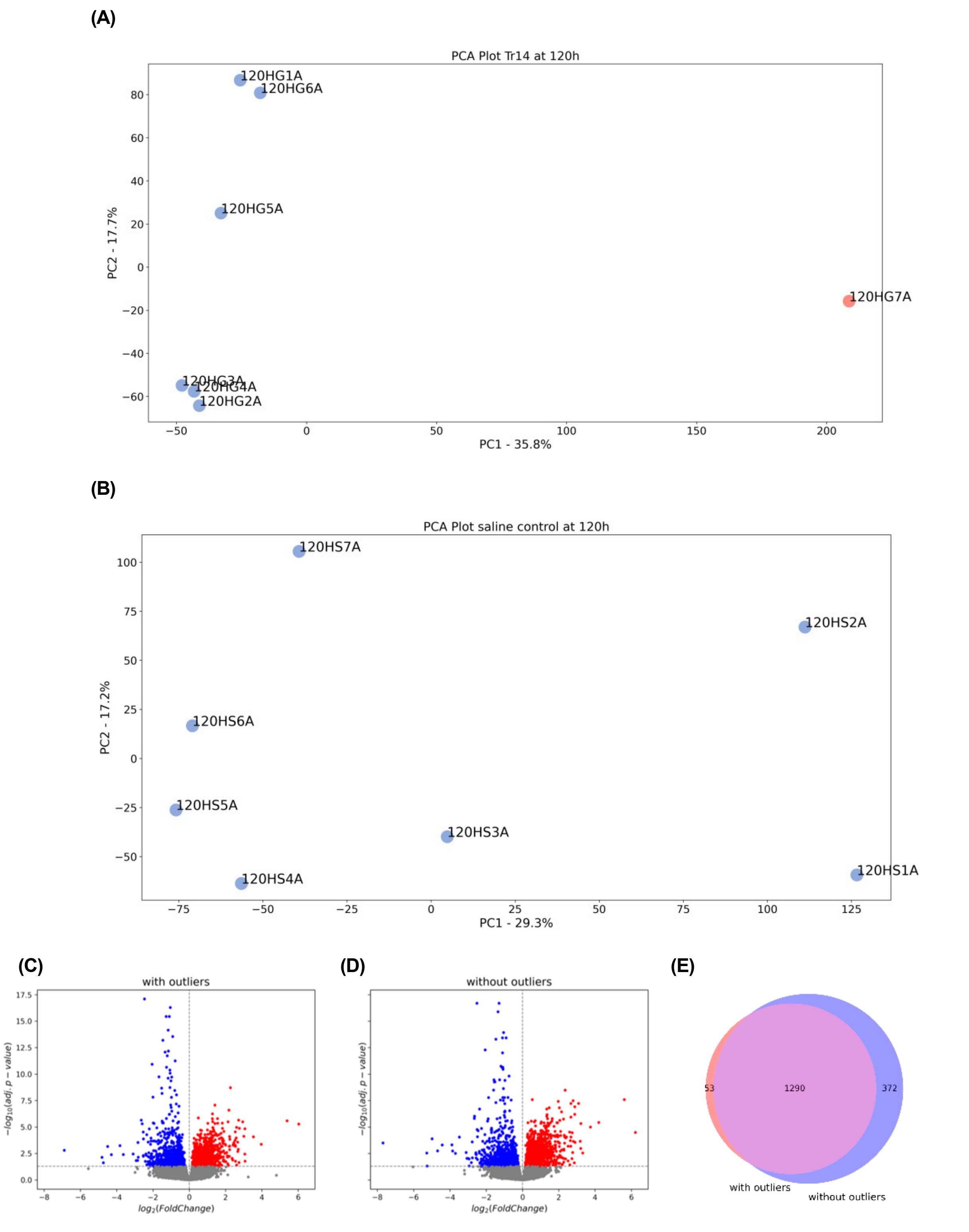

Figure S4: PCA analysis of RNA-seq samples for Tr14 treatment (A) and saline control (B) at 120h. Outliers (orange) were removed afterward. (C and D) Volcano plots showing the impact of outlier removal on the DeSeq2 differential analysis for Tr14 vs. control. (E) Venn diagram comparing the number of significant genes (adj. *p*-value < 0.05) before and after the removal of outliers.

Diclofenac vs. topical control at 96h

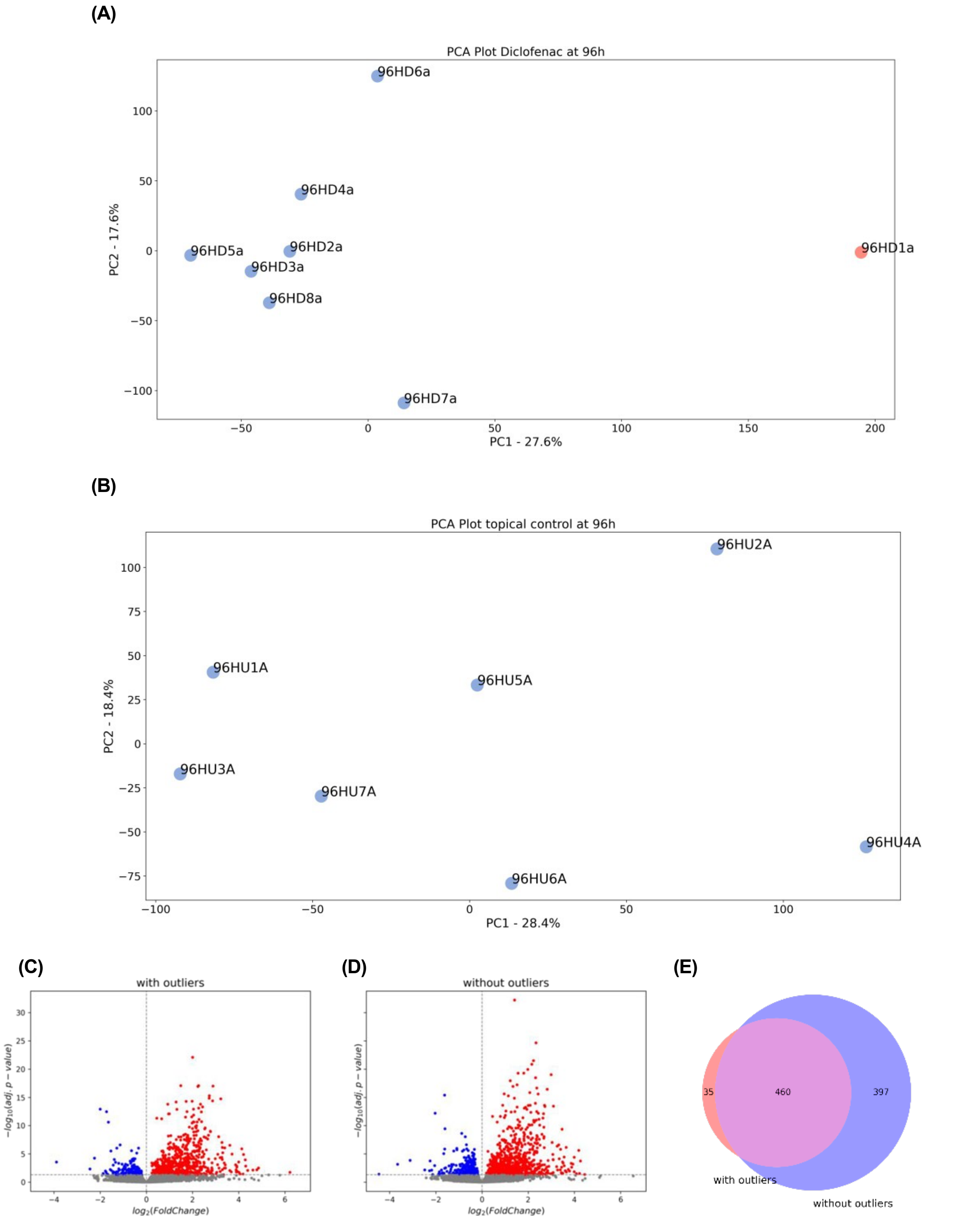

Figure S5: PCA analysis of RNA-seq samples for diclofenac treatment (A) and placebo control (B) at 96h. Outliers (orange) were removed afterward. (C and D) Volcano plots showing the impact of outlier removal on the DeSeq2 differential analysis for diclofenac vs. control. (E) Venn diagram comparing the number of significant genes (adj. *p*-value < 0.05) before and after the removal of outliers.

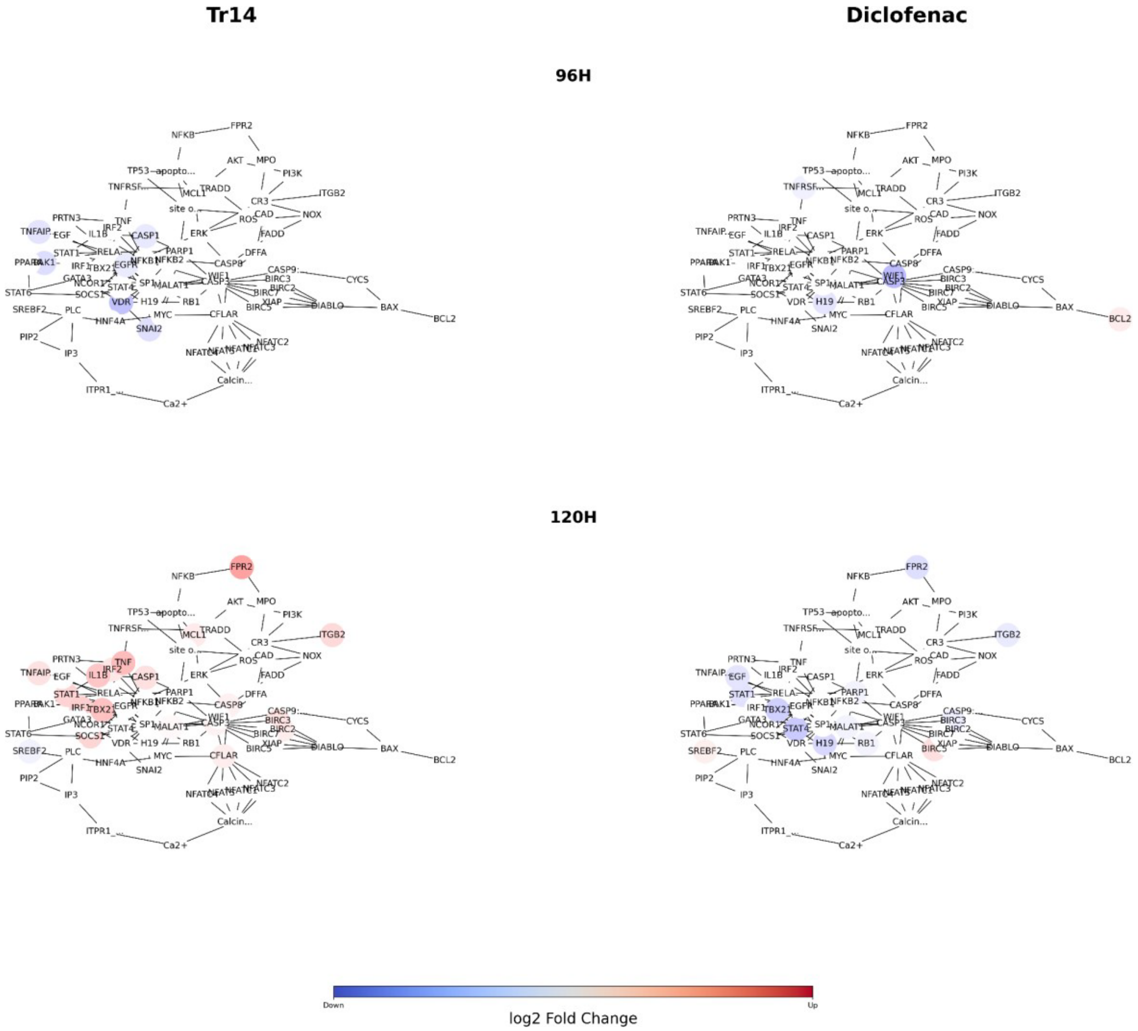

Figure S6: Core Regulatory Networks (CRNs) of the "apoptotic process" phenotype for Tr14 and Diclofenac treatment at 96 and 120 hours. Gene triplets in the molecular interaction map connected to the phenotype are ranked by *log*2 fold change values and network features. The highest ranked motifs were than selected and merged into the CRNs.

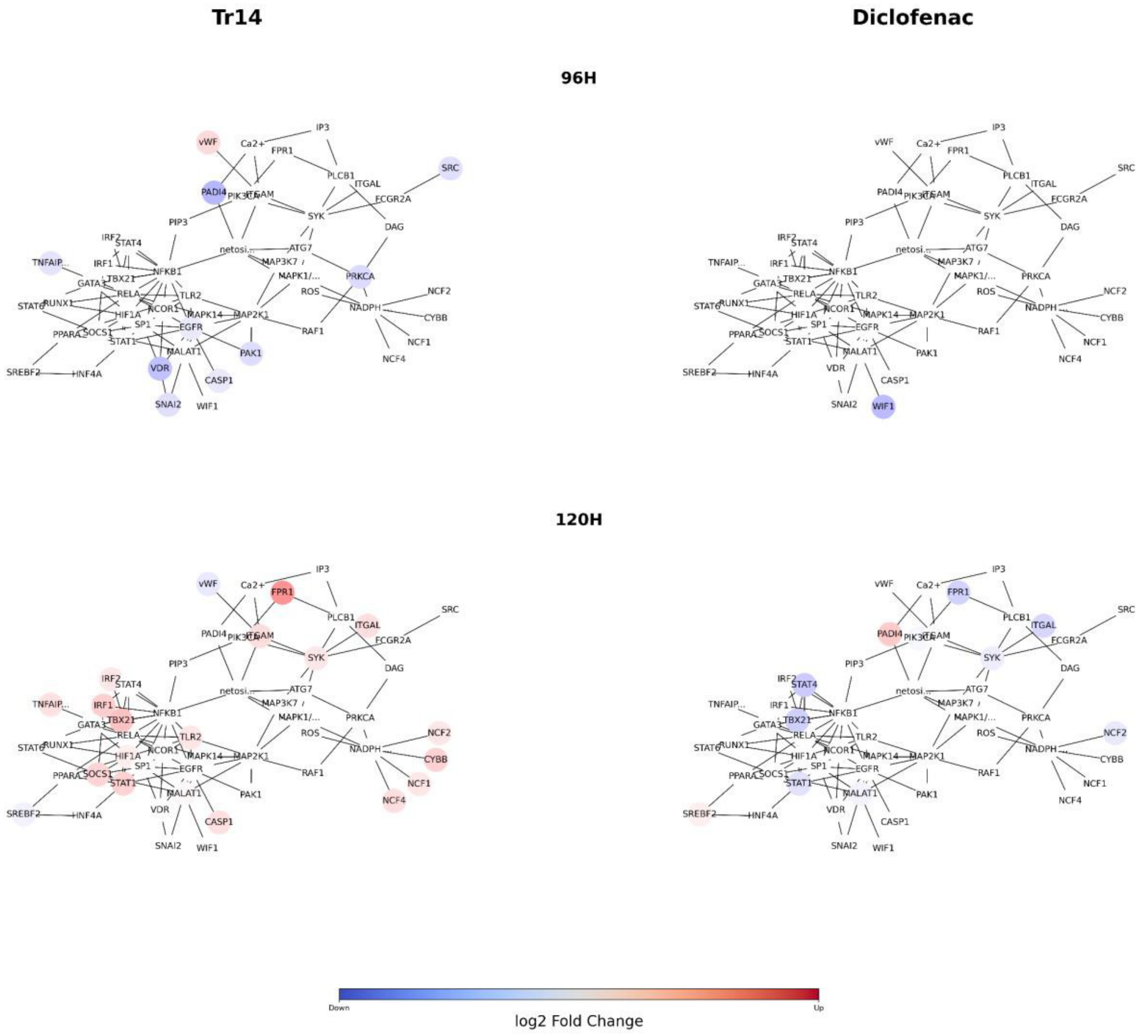

Figure S7: Core Regulatory Networks (CRNs) of the "NETosis" phenotype for Tr14 and Diclofenac treatment at 96 and 120 hours. Gene triplets in the molecular interaction map connected to the phenotype are ranked by *log*2 fold change values and network features. The highest ranked motifs were than selected and merged into the CRNs.

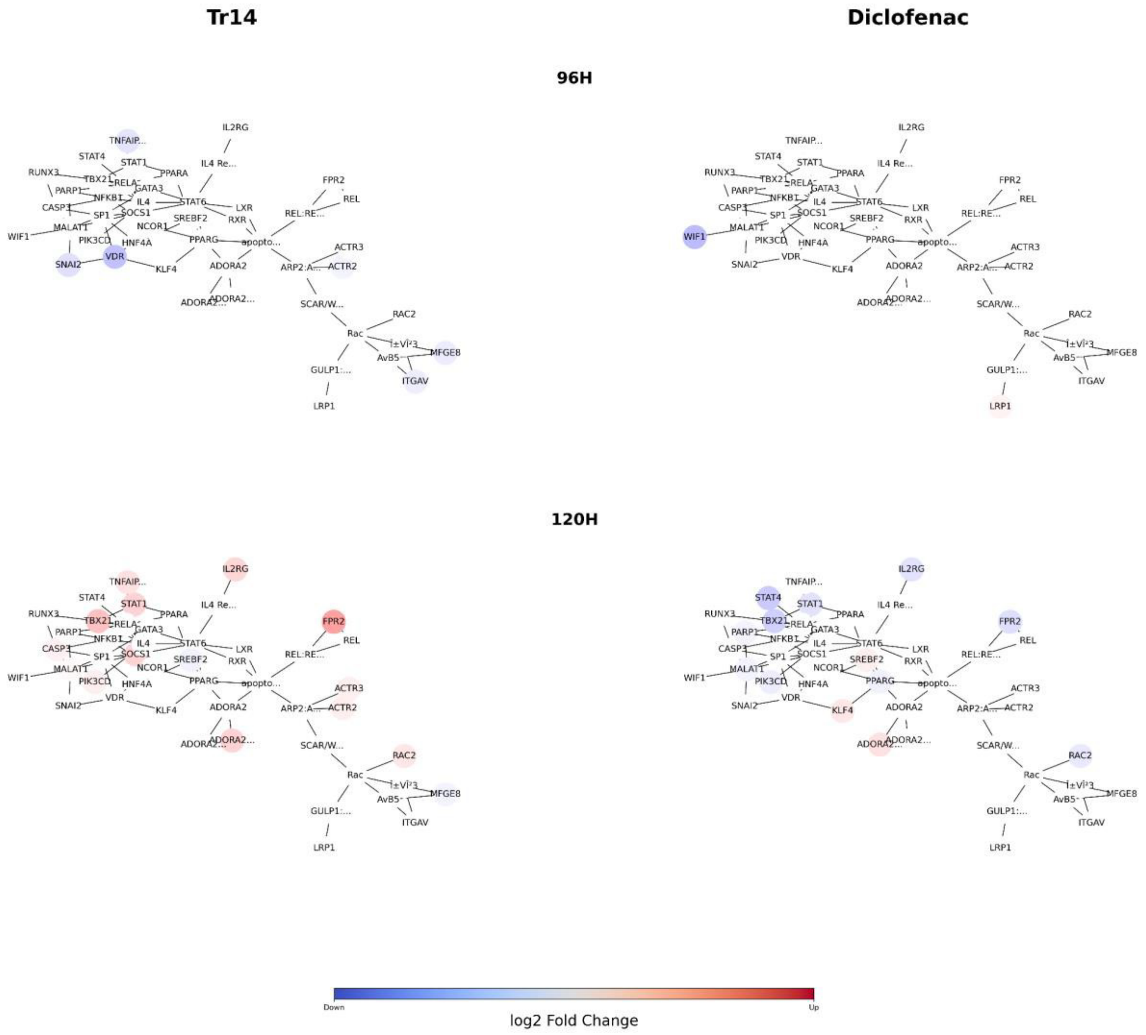

Figure S8: Core Regulatory Networks (CRNs) of the “apoptotic cell clearance” (efferocytosis) phenotype for Tr14 and Diclofenac treatment at 96 and 120 hours. Gene triplets in the molecular interaction map connected to the phenotype are ranked by *log*2 fold change values and network features. The highest ranked motifs were than selected and merged into the CRNs.

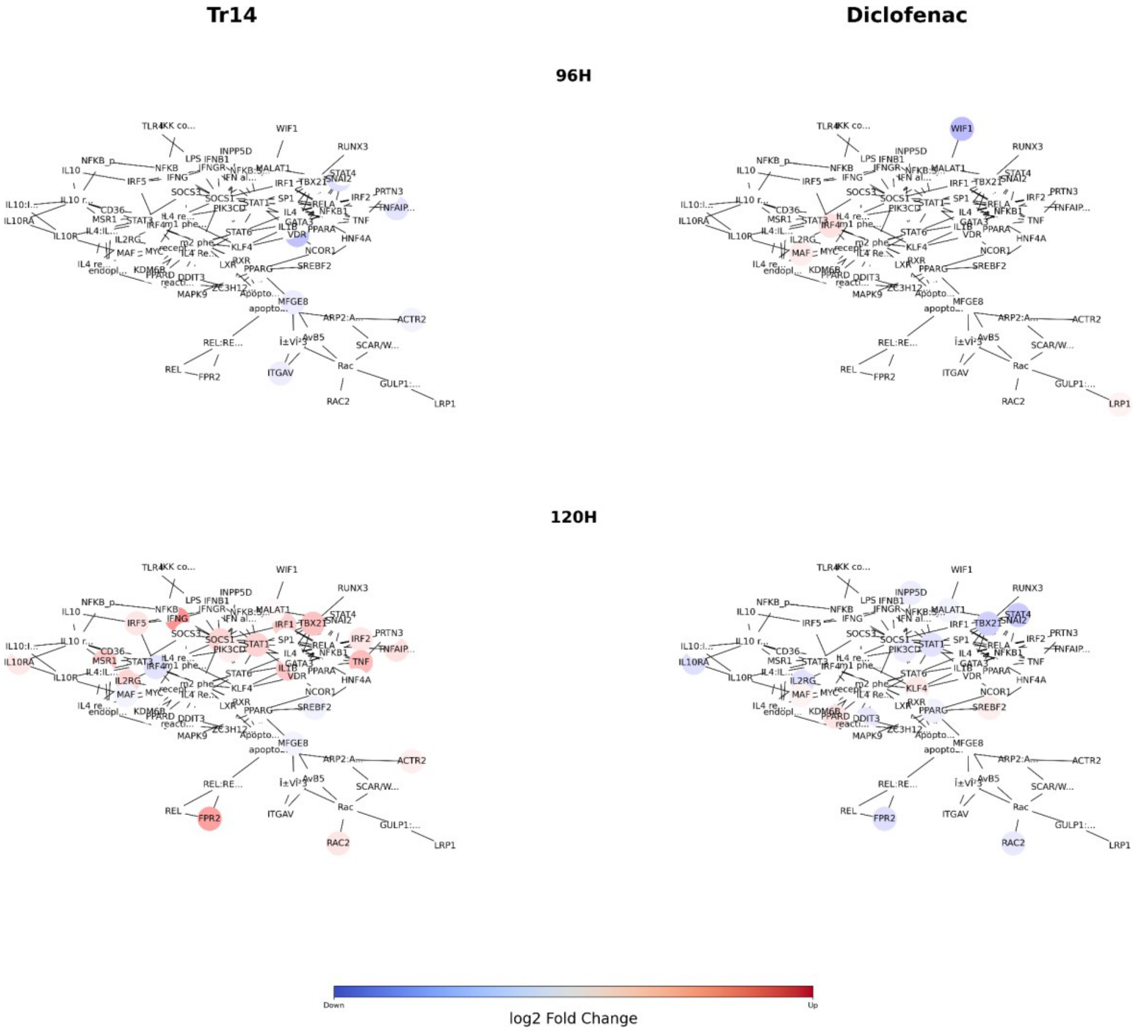

Figure S9: Core Regulatory Networks (CRNs) of the “M2 phenotype and behavior” phenotype for Tr14 and Diclofenac treatment at 96 and 120 hours. Gene triplets in the molecular interaction map connected to the phenotype are ranked by *log*2 fold change values and network features. The highest ranked motifs were than selected and merged into the CRNs.
